# Non-steroidal Anti-inflammatory Drugs Are Unlikely to Inhibit Radiographic Progression of Ankylosing Spondylitis: A Systematic Review

**DOI:** 10.3389/fmed.2019.00214

**Published:** 2019-10-04

**Authors:** Jing-Ru Zhang, Dan-Dan Pang, Sheng-Ming Dai

**Affiliations:** ^1^Department of Rheumatology & Immunology, First Affiliated Hospital of Zhejiang Chinese Medical University, Hangzhou, China; ^2^Department of Rheumatology & Immunology, Changhai Hospital, Second Military Medical University, Shanghai, China; ^3^Department of Rheumatology & Immunology, Shanghai Jiao Tong University Affiliated Sixth People's Hospital, Shanghai, China

**Keywords:** ankylosing spondylitis, non-steroidal anti-inflammatory drugs (NSAIDs), radiographic progression, structural damage, drug treatment

## Abstract

**Objective:** To clarify if non-steroidal anti-inflammatory drugs (NSAIDs) could retard the disease progression of ankylosing spondylitis (AS).

**Methods:** A systematic search of Embase, Pubmed, and the Cochrane Central Register of Controlled Trials (CCRCT) databases was conducted. Structural damage of AS was evaluated using spinal radiographs to assess modified Stoke Ankylosing Spondylitis Spine Score (mSASSS).

**Results:** Five full-text papers (from 2 prospective and 2 retrospective studies) were included. Of the 4 studies deemed relevant, 3 reported no significant inhibition of spinal progression in AS patients treated continuously with NSAIDs, as determined by radiograph over 2–3 years. Only the 1st prospective randomized trial demonstrated that 2-year continuous use of celecoxib reduced mean changes in mSASSS of AS patients compared with on-demand treatment. However, the dosage difference of celecoxib between the two groups in the study seemed to be too small to elicit such differences in radiographic progression, while the therapy did not elicit any differences in disease activity, C-reactive protein (CRP) levels or global pain. Of the 3 studies that reported radiographic progression in the subgroup with elevated CRP, only *post-hoc* analysis of the 1st randomized study revealed that the patients treated continuously with NSAIDs had less radiological progression than those using on-demand NSAIDs. In 2 studies that reported radiographic progression in the patient subgroup with baseline syndesmophytes, both reported that there was no significant inhibition of progression of mSASSS in patients who had received continuous NSAID treatment compared with patients given on-demand NSAIDs.

**Conclusion:** The available evidence suggests that NSAIDs are unable to delay radiographic progression of AS even in patients with elevated CRP levels.

## Introduction

Ankylosing spondylitis (AS) is a chronic form of inflammatory arthritis that most often affects the spine, and finally results in loss of mobility and function. This disorder is characterized by syndesmophytes that form along the rim of the vertebral bodies and ankylosis of the spine (1, 2). The primary goal of treatment for AS patients is to optimize the long-term quality of life by reducing the degree of inflammation, as well as delaying structural changes of the disease. However, it is not sufficient to retard radiographic progression of spine by resolving inflammation, which might be achieved by treatment with TNF-α inhibitors (TNFi) (3). Non-steroidal anti-inflammatory drugs (NSAIDs) are recommended as the initial drug treatment for AS patients by several academic organizations including the Assessment of SpondyloArthritis International Society (ASAS)/European League Against Rheumatism (EULAR) and American College of Rheumatology (ACR)/Spondylitis Association of America/Spondyloarthritis Research and Treatment Network (SPARTAN) (4, 5), mainly because both non-selective cyclooxygenase (COX) inhibitors and selective COX-2 antagonists are effective in relieving symptoms in patients with AS that is active (6). There is some conflicting evidence as to whether or not long-term treatment with NSAIDs delays the development of damage to the spine (7–13). The aims of this review were to analyze the background, methodology employed and findings of published studies to determine whether NSAIDs possess disease-modifying properties in patients with AS.

## Methods

### Search Strategy

We searched the following databases from inception to Feb. 2019: MEDLINE, EMBASE, the Cochrane Central Register of Controlled Trials (Supplementary Material), as well as additional resources including the Database of Abstracts of Review of Effects, Scopus for conference proceedings, and clinical trial registries for ongoing and recently finished studies. In order to retrieve additional references, we also carried out manual searches of the bibliography references cited in each included article.

### Inclusion and Exclusion Criteria

We considered all randomized controlled trials (RCT), quasi-RCT (i.e., where allocation was not truly random), and observational studies in English would be included without restriction of publication type. We included trials of adults (>18 years old, but with no upper age limit) who met the modified New York criteria for AS (14). We included studies comparing NSAID in all possible variations (dosage, intensity, mode, duration, or timing of delivery) to placebo, no therapy, another NSAID, other pharmacological therapy, non-pharmacological therapy, combination therapy, different doses or modes of delivery, or frequency or duration. Only studies that were published as full articles or were available as a full trial report would be included. Studies which did not concerned about AS patients and were not relevant to spinal radiographic progression were excluded. Editorials, review articles, letters, case reports, opinions, author reply, or comments were also excluded.

### Study Selection and Data Collection

Two review authors (JR and DD) independently screened titles and abstracts, and full-text papers if necessary to determine inclusion. If any disagreement occurs, a decision will be made through discussion or consultation with a third author (SM). Data extraction was performed by the same authors (JR and DD) using a standardized data extraction form. The following data were extracted: main characteristics of study (authors, year of publication), study design, number of included patients, baseline characteristics of AS patients, usage pattern of NSAIDs, value of radiographic progression, and the relevant outcome data. If the reviewers have different opinions, the issue will be resolved through discussion or consultation with a third author (SM).

### Assessment of Risk of Bias

Two review authors (JR and DD) independently assessed risk of bias of each study using the Cochrane risk of bias tool (RoB 2.0) (Supplementary Table 1). This tool involves RoB assessment in five domains (randomization process, deviations from the intended interventions, Missing outcome data, measurement of the outcome, selection of the reported result). Each domain was judged as “low risk of bias,” “high risk of bias,” or “some concerns.” Discrepancies between reviewers were solved by discussion; a third reviewer (SM) was available in case no consensus could be achieved.

## Results

### Characteristics of Included Studies

**Figure 1:**
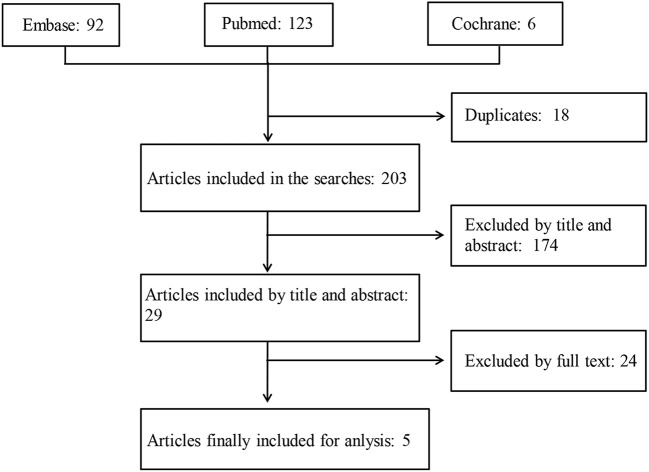
A schematic illustration showing the selection process for articles included in the systemic review. The MEDLINE (via Pubmed), EMBASE (via Ovid), and Cochrane databases were searched using specified terms, to retrieve the articles analyzing the long-term effects of non-steroidal anti-inflammatory drugs on structural damage in patients with ankylosing spondylitis.

A total of 221 articles were retrieved, 18 of them were duplicates, and 195 failed to meet the inclusion criteria. Two meeting abstracts lacking full texts (12, 13) and one paper reporting protocols comparing the effects of treatment with NSAIDs combined with TNFi vs. TNFi alone on spinal radiographic progression over a period of 2 years (15) were further excluded. Finally, 5 full-text papers (7–11) were included in this systemic review (Figure 1). The results of 5 full-text papers were derived from 2 prospective randomized controlled trials and 2 observational cohort studies (Table 1).

**Table 1:** Baseline characteristics of AS patients with complete sets of radiographs in the enrolled studies.

**Study**	**NSAIDs**	**Patients (*n*)**	**Age (years)**	**Male (%)**	**Disease duration (years)**	**Positive HLA-B27 (%)**	**BASDAI**	**BASFI**	**CRP (mg/L)**	**ESR (mm/h)**	**mSASSS**
Wanders et al. (7) Kroon et al. (8) Prospective controlled trial	Continuous	76	40.9 ± 9.8	66	13.0 ± 10.2	88.2	NA	3.0 ± 2.3	13.1 ± 15.3	16.5 ± 13.1	7.9 ± 14.7
	On-demand	74	37.9 ± 11.9	70	10.2 ± 9.3	87.8	NA	3.6 ± 2.8	12.2 ± 17.5	17.5 ± 18.1	9.3 ± 15.2
Sieper et al. (9) Prospective controlled trial	Continuous	62	40.7 ± 9.6^*^	71	12.8 ± 11.3^*^	88.7	4.1 ± 1.5	2.9 ± 2.1^*^	7.8 ± 7.4	NA	10.9 ± 15.5
	On-demand	60	45 ± 10.4	67	17.0 ± 12.6	91.7	4.2 ± 1.5	3.7 ± 2.2	12.5 ± 15.1	NA	16.4 ± 18.2
Poddubnyy et al. (10) Retrospective cohort	Index ≥ 50^#^	24	38.7 ± 9.8	67	5.5 ± 2.7	79.2	4.7 ± 2.1^*^	4.1 ± 2.1^**^	7.9 ± 8.7	15.8 ± 9.2	6.7 ± 7.7
	Index <50^#^	64	36.2 ± 12.4	67	5.0 ± 2.9	85.9	3.5 ± 2.1	2.4 ± 2.2	11.7 ± 12.3	21.7 ± 19.6	5.7 ± 11.6
Park et al. (11) Retrospective cohort	NSAID^&^	80	34.4 ± 11.9	76.2	4.1 ± 2.9	88.7	3.2 ± 1.6^**^	NA	11 ± 13^**^	NA	7.3 ± 10.8
	TNFi^&^	135	32.8 ± 11.5	81.5	4.3 ± 2.7	88.1	6.7 ± 1.6	NA	22 ± 27	NA	6.2 ± 9.9

*AS, ankylosing spondylitis; BASDAI, Bath Ankylosing Spondylitis Disease Activity Index; BASFI, Bath Ankylosing Spondylitis Functional Index; BASRI, Bath Ankylosing Spondylitis Radiology Index; CRP, C reactive protein; ESR, erythrocyte sedimentation rate; HLA, human leukocyte antigen; mSASSS, modified Stoke Ankylosing Spondylitis Spine Score; NA, not available; NSAIDs, non-steroidal anti-inflammatory drugs; TNFi, TNF-α inhibitors*.

* P < 0.05 and

** *P < 0.01 compared with low NSAIDs intake group*;

# *An index (range 0~100) of NSAIDs intake was calculated, based on both dose and duration/regiment of drug intake (0 = no NSAIDs intake at all; 100 = daily NSAIDs intake in a dose equivalent to diclofenac 150 mg over the whole period of interest)*.

& *The data were derived from 2 independent Korean cohorts of early AS with inflammatory back pain <10 years from initial onset. The NSAID index for the NSAID group was 46.3 ± 23.3 during 0–2 year interval and 42.0 ± 24.2 during 2–4 year intervals, while for the TNFi group it was 23.3 ± 23.6 during both 0–2 and 2–4 year intervals. Values are mean ± SD if not otherwise specified*.

The article by Wanders et al. (7) and the other one by Kroon et al. (8) adopted the same one prospective trial (namely the 1st prospective trial). In this trial, paired radiographs were available for 76 out of 111 (68.5%) patients in the group treated continuously with NSAIDs and for 74 out of 104 (71.8%) patients in the NSAID on-demand group, although 215 patients with AS were randomly allocated into the two groups at the beginning of the trial (7, 8). In the 2nd prospective trial, 62 out of 85 (72.9%) patients in the group treated continuously and 60 out of 82 (73.2%) patients in the group who given drugs on-demand successfully completed the clinical research investigation (9). Baseline characteristics of patients with AS who had a complete set of radiographs from all the five studies are shown in Table 1. In the retrospective study (10), the baseline activity of AS was slightly less than in the controls. Younger age, a shorter duration of AS and a better functional index at baseline in the continuously treated arm were noted in the prospective study of Sieper et al. (9). To evaluate radiographic progression, all the selected studies assessed the modified Stoke Ankylosing Spondylitis Spine Score (mSASSS).

The treatment was classified into two groups, namely continuous therapy vs. on-demand therapy with NSAIDs for a duration of 2–3 years in 3 studies, or a high NSAID index (≥50) vs. a low NSAID index (<50) in a retrospective study. According to the recommendation by the ASAS, the NSAID index was calculated according to both dosage and the duration of drug intake (100 means an intake of drug equivalent to 150 mg/d of diclofenac over the whole duration of investigation, and 0 means no NSAID intake ever) (16). One observational study compared two independent cohorts of Korean patients with recently diagnosed AS, who were treated with NSAIDs alone (conventional group) or TNFi (with or without NSAIDs) for over 4 years (11).

### Continuous Treatment With NSAIDs Fails to Delay Radiographic Progression in Most AS Patients, Especially Those With Normal C-Reactive Protein (CRP) Levels at Baseline

Table 2 shows that 3 out of 4 studies revealed no significant inhibition of spinal radiographic progression in AS patients treated by continuous NSAIDs (or high NSAID intake) over 2–4 years. In the 1st prospective randomized trial, Wanders et al. (7) reported that 2-year continuous use of NSAIDs decreased mean changes in mSASSS (ΔmSASSS) of AS patients, compared to the on-demand NSAID group (Table 2). Rapid radiographic progression (ΔmSASSS ≥ 3 units) during 24 months occurred less frequently in the continuously treated group compared to the group of patients who were given NSAIDs on-demand (11% vs. 23%). The mean celecoxib dose was 201 ± 93 mg/d in the on-demand group vs. 243 ± 59 mg/d in the continuously dosed group of patients. It should be pointed out that the average dose of celecoxib administered each day to the two groups of patients was as small as 42 mg (95% CI: 21–63).

**Table 2:** Radiographic progression in the AS patients with complete sets of radiographs after long-term treatment with NSAIDs.

**Study**	**NSAIDs**	**Patients (*n*)**	**Follow-up (years)**	**Δ mSASSS (mean ± SD)**	***P*-value**
Wanders et al. (7) Prospective controlled trial	Continuous	76	2	0.4 ± 1.7	0.002
	On-demand	74	2	1.5 ± 2.5	
Sieper et al. (9) Prospective controlled trial	Continuous	62	2	1.28 (0.7–1.9) ^a^	0.39
	On-demand	60	2	0.79 (0.2–1.4) ^a^	
Poddubnyy et al. (10) Retrospective cohort	Index ≥ 50	24	2	0.02 ± 1.39	0.142
	Index <50	64	2	0.96 ± 2.78	
Park et al. (11) Retrospective cohort	NSAID	80	>4	NA	
	TNFi	135	>4	NA	

*AS, ankylosing spondylitis; ΔmSASSS, changes in modified Stoke Ankylosing Spondylitis Spine Score; n, number; NA, not available; NSAIDs, non-steroidal anti-inflammatory drugs; SD, standard deviation; TNFi, TNF-α inhibitors*.

a *95% Confidence intervals*.

In the other prospective randomized trial, Sieper et al. (9) found that significant mSASSS progression occurred in both the continuous NSAID and on-demand NSAID patient groups. Different from the results of a previous trial, the change in mSASSS in the continuously dosed patient group appeared to be slightly greater than in the on-demand group, but the difference was not statistically different (*P* = 0.39, Table 2). Nevertheless, the BASDAI values decreased to 2.7 in the group of patients treated continuously with NSAIDs and was 3.2 in the on-demand group over 2 years, which confirmed that patients in the continuously treated group received, overall, a higher dosage of NSAIDs. Over 2 years, the mean NSAID index was 76 for the continuously treated group and 44 for the on-demand group of patients. The difference in the dosage of diclofenac administered each day to the two groups was 112 mg vs. 66 mg, respectively. Changes in mSASSS were 0.7 and 1.2 units in patients with an NSAID index <50 (*n* = 65) and with an index ≥50 (*n* = 94), respectively (*P* > 0.05). Furthermore, in patients whose NSAID index was ≥75 (*n* = 53) and <25 (*n* = 23), mSASSS changes over 2 years were 0.5 and 1.1 units, respectively (*P* > 0.05). All these data confirmed that a high NSAID intake did not prevent structural damage in AS patients.

In a retrospective analysis (data from the GESPIC patients), although Poddubnyy et al. (10) reported that a high NSAID intake was associated with delayed radiographic spinal progression over a 2-year period. Actually, there was a trend for decreased mSASSS changes in patients taking high doses of NSAIDs, but the data was not statistical significantly different (*P* = 0.142) (Table 2). Over 2 years, the NSAID intake index was 33.7 ± 28.0 in these AS patients. In patients with an NSAID intake index < 33, 33–65 or ≥ 66, changes in mSASSS were 0.86 ± 2.93, 0.75 ± 1.84 and 0.10 ± 1.70 units, respectively, over 2-years (*P* = 0.587 for all three groups). A limited number of AS patients with low NSAID intake showed definite spinal radiographic progression, characterized as an increase in mSASSS ≥ 2 units over a 2 year period, compared with patients on a high NSAID intake: 21.9% (*n* = 2) vs. 8.3% (*n* = 14), respectively. However, the apparent difference was not statistically significant (*P* = 0.142). Furthermore, the dosage of NSAID consumption had no influence on the rate of spinal radiographic progression in those patients diagnosed with non-radiographic axial spondyloarthritis (10).

In the Korean cohorts, although mSASSS changes in the NSAID group, or the TNFi group and their subgroups were not directly reported, NSAID intake indices of both groups were not related to mSASSS changes, while TNFi inhibited mSASSS change over a 2-year time scale (11).

Only 2 studies reported radiographic progression in the subgroup without elevated acute phase reactants (Table 3). Both studies found that continuous treatment with NSAIDs did not inhibit radiographic progression in AS patients who had normal levels of CRP (8, 10).

**Table 3:** Changes in mSASSS of the AS patients stratified with baseline CRP levels after 2-year treatment with NSAIDs.

**Study**	**CRP level**	**NSAIDs**	**Patients (*n*)**	**Δ mSASSS (mean ± SD)**	***P*-value**
Kroon et al. (8) Prospective controlled trial	CRP > 5 mg/L	Continuous	52	0.2 ± 1.6	0.003
		On-demand	45	1.7 ± 2.8	
	CRP <5 mg/L	Continuous	21	0.9 ± 1.8	0.62
		On-demand	25	0.8 ± 1.1	
Sieper et al. (9) Prospective controlled trial	CRP > 5 mg/L	Continuous	34	1.68 (0.7–2.6)^a^	0.28
		On-demand	35	0.96 (0.0–1.9)^a^	
Poddubnyy et al. (10) Retrospective cohort	CRP > 6 mg/L	Index ≥ 50	13	0.0 ± 1.41	0.11
		Index <50	32	1.69 ± 3.48	
	CRP <6 mg/L	Index ≥ 50	11	0.05 ± 1.41	0.54
		Index <50	32	0.23 ± 1.58	
	CRP > 6 mg/L + syndesmophytes	Index ≥ 50	7	0.14 ± 1.80	0.02
		Index <50	11	4.36 ± 1.53	

*AS, ankylosing spondylitis; CRP, C-reactive protein; ΔmSASSS, changes in modified Stoke Ankylosing Spondylitis Spine Score; n, number; NSAIDs, non-steroidal anti-inflammatory drugs; SD, standard deviation*.

a *95% Confidence intervals*.

### No Definitive Evidence Supports the View That Continuous NSAID Treatment Delays Radiographic Progression in AS Patients With High CRP Levels

Recently, it has been demonstrated that an elevated serum CRP level is an independent indicator for radiographic progression of sacroiliitis (17) and of spinal (18). Three studies reported radiographic progression in a subgroup of AS patients whose CRP levels were elevated (Table 3). Only the *post-hoc* analyses of the 1st randomized trial with celecoxib showed that patients with elevated CRP levels (>5 mg/L), who received continuous NSAIDs, had less radiological progression than patients with elevated CRP levels taking on-demand NSAIDs (8).

In the other 2 studies, high NSAID intake did not inhibit radiological progression in AS patients with elevated CRP levels compared to low NSAID intake (Table 3). Poddubnyy et al. (10) reported that high NSAID intake had a protective impact only in those patients with both elevated CRP and syndesmophytes compared with low NSAID intake. However, the patient numbers in the subgroup with both elevated CRP and syndesmophytes were rather small (7 vs. 11 patients) (Table 3).

### Continuous NSAID Treatment Fails to Delay Radiographic Progression in Patients With Syndesmophytes

Many researchers have found that baseline syndesmophytes present the greatest risk for spinal radiographic progression in AS patients (18–21). Only 2 studies reported radiographic progression in a subgroup with baseline syndesmophytes (Table 4). Both of them demonstrated that there was no further mSASSS progression in AS patients with baseline syndesmophytes treated with on-demand NSAIDs compared with continuous treatment. The patient number in each subgroup was also small, especially in the study of Poddubnyy et al. (10).

**Table 4:** Changes in mSASSS of the AS patients with syndesmophytes at baseline after 2-year treatment with NSAIDs.

**Study**	**NSAIDs**	**Patients (*n*)**	**ΔmSASSS (mean ± SD)**	***P*-value**
Sieper et al. (9) Prospective controlled trial	Continuous	33	2.11 (1.1–3.1)^a^	0.10
	On-demand	37	0.95 (0.0–1.9)^a^	
Poddubnyy et al. (10) Retrospective cohort	Index ≥ 50	11	0.09 ± 1.80	0.076
	Index <50	17	2.74 ± 4.58	

*AS, ankylosing spondylitis; ΔmSASSS, changes in modified Stoke Ankylosing Spondylitis Spine Score; n, number; NSAIDs, non-steroidal anti-inflammatory drugs; SD, standard deviation*.

a *95% Confidence intervals*.

## Discussion

The chronic debilitating condition, AS, is characterized by enthesitis and axial skeletal ankylosis. The relationship between the formation of new bone and local inflammation in AS patients has been strongly debated since the first data were obtained on radiographic progression in patients who received TNFi therapy (22). The mechanism of action of NSAIDs is to inhibit the activity of COX enzymes, which catalyze the production of prostaglandins that influence inflammation. Our literature review identified that most of the studies challenge the earlier concept that NSAIDs delay radiographic progression in AS. In two meeting abstracts, Schiotis et al. (13) reported both the two treatment groups for AS showed significant radiographic progression over 3 years (*P* < 0.001), while continuous NSAIDs (*n* = 81) did not inhibit radiographic progression; and Haroon et al. (12) reported there was no significant difference between the changes in mSASSS over 2 years compared continuous NSAIDs in combination with TNFi and TNFi alone in 40 AS patients. Multivariable analysis of data from both OASIS and Swiss cohorts of patients, with ≥10 years follow-up, revealed that NSAID use was not an independent factor related to radiographic progression (21, 23). All these data further support the view that NSAIDs may be unable to delay radiographic progression of AS. Before drawing an appropriate conclusion, the following issues should be considered.

### Inflammation I*s* Related to New Bone Formation in AS

Data from magnetic resonance imaging, histopathology and treatment interventions indicate that the disorder begins with inflammation, then bone erosion, followed by replacement of fat metaplasia and new bone formation (24). Persisting high disease activity caused by inflammation, contributes to accelerated spinal radiographic progression in AS (25, 26). Data from earlier clinical trials indicated that therapy with TNFi for ≤2 years failed to slow the progression of spinal structural damage, in comparison with TNFi-naïve groups (3). Observational studies, however, have shown that earlier initiation of TNFi and long-term TNFi therapy may reduce radiographic progression (23, 27, 28).

Increasing evidence supports the view that early and effective anti-inflammatory therapy is vital for inhibition of ongoing ankylosis. Many researchers have found that the presence of baseline syndesmophytes presents the greatest risk for spinal radiographic progression (3), indicating that the patients with advanced AS might not benefit from therapeutic intervention to prevent structural damage. In conflict with this later evidence, Poddubnyy et al. (10) demonstrated a protective effect of high dose NSAIDs, only in patients who had both increased CRP levels and syndesmophytes, but not in patients with only elevated CRP levels, compared with low NSAID intake. It is very difficult to explain the findings by Poddubnyy et al.

### Inhibition of Inflammation by NSAIDs Is Limited in AS

NSAIDs are very effective in relieving pain and stiffness in AS patients. However, inflammation might persist even when symptoms are well-controlled. In a recently published paper, after 4-week optimal NSAID treatment, nearly half (44%) of the initially active AS patients still had a BASDAI score ≥ 4, in spite of the fact that an improvement in ASAS40 was observed in 35% of them. Moreover, CRP levels, Berlin MRI scores of sacroiliac joints, and the ratio of individuals with a positive MRI finding did not significantly improve after 4-week treatment with NSAIDs (29).

After 6-week's treatment with 90 mg of etoricoxib daily, an ASAS20 response was found in 60% of patients. However, only 13 of 60 active axial inflammatory lesions at baseline were improved, while 5 lesions worsened in spite of etoricoxib treatment (30). In the Korean cohorts of AS patients, NSAIDs were not effective in decreasing CRP levels (11). On the other hand, NSAIDs were also reported to be effective in decreasing CRP levels after 12-weeks treatment (31). In the 1st trial demonstrating the protective effect of continuous NSAIDs, no significant changes were shown in CRP levels (*P* = 0.82), BASDAI (*P* = 0.51), global pain (*P* = 0.44) or patient global evaluation (*P* = 0.94) after 2 years treatment with continuous or on-demand celecoxib (7). These data obviously showed that the slower radiographic progression of continuous NSAIDs than on-demand NSAIDs can not be attributed to its greater inhibition of inflammation or better control of disease activity in this trial, although elevated CRP levels and disease activity are well-demonstrated to be the two major risk factors related to axial radiographic progression of AS in other studies. Because NASIDs only possess very limited anti-inflammatory efficacy in AS patients, it seems that NSAIDs are unlikely to slow structural damage by anti-inflammation.

### Effects of Prostaglandins on Bone Remodeling Are Complex

It is well-known that NSAIDs exert their pharmacological actions through inhibition of COX activity and prostaglandin biosynthesis. Although NSAIDs have been reported to inhibit bone formation related to fracture healing (32, 33), the effects of prostaglandins (PGs) on bone remodeling are complex, since PGs can stimulate both bone resorption and bone formation (34). For example, prostaglandin E2 (PGE2) potently induces bone resorption by increasing the RANKL/OPG ratio to stimulate osteoclast differentiation. PGE2 also induces proliferation and differentiation of osteoblasts to stimulate bone formation. On the other hand, prostaglandins also have inhibitory effects on fully differentiated osteoblasts and osteoclasts (35). For example, PGE2 inhibits collagen synthesis and matrix mineralization by osteoblasts (36). To date, the exact role of prostaglandins in bone metabolism at the site of enthesitis remains unclear, let alone the net effect of prostaglandin biosynthesis inhibition by NSAIDs on the formation of new bone in AS patients. Based on the available evidence, we can't expect that NSAIDs could inhibit syndesmophyte formation in AS by inhibiting the synthesis of PGE2.

### Dosage Differences of Administered NSAIDs Should B*e* Concerned

All studies just compared continuous and on-demand NSAIDs therapy or compared high and low NSAID intake treatment in AS patients. However, an index ≥ 50, which takes both dose and days of use into account, was not the same when treatment was continuous. Up to now, there have been no prospective randomized placebo-controlled clinical trials that have specifically studied the efficacy of NSAID therapy on radiographic progression in patients with AS, that have yielded definitive data. In fact, to perform such a placebo-controlled trial is not plausible.

It is very difficult to imagine that as small difference as 42 mg/d in the mean dose of celecoxib could result in such a significant protection of structural damage in AS patients (7, 8). The underlying mechanisms were not explored. Nearly 30% of patients were excluded from the analysis because of incomplete sets of radiographs, and therefore selection bias cannot be ruled out.

In the 2nd randomized trial (9), the magnitude of differences in the mean daily doses of diclofenac in the continuous (112.5 mg daily) and demand (66 mg daily) groups of patients was 68.5 mg over 2 years, which was greater than that of celecoxib in previous reports (7, 8). The null effect of continuous diclofenac on the formation of new bone in this trial (9) could not be attributed to the dose of NSAIDs. The mSASSS increased from 12.1 to 12.6 (difference 0.5) in those patients with an NSAID index <25 vs. from 12.0 to 13.1 (difference 1.1) in those patients with an NSAID index ≥ 75 (9), further demonstrating the null effect of higher dose NSAIDs on the formation of new bone. At the therapeutic dosages administered, all NSAIDs are able to block COX-2 activity to the same degree (37), so the lack of efficacy of continuous diclofenac on radiographic progression could not be attributed to the selective activity on COX-2 either.

### Reliability of Assessment of Spinal Radiographic Progression Is Limited in AS

Although there are some radiographic scoring methods, the mSASSS has been used most frequently in recent studies (38). In mSASSS, erosions or sclerosis, squaring (score 1), syndesmophytes (score 2), or bridged syndesmophytes (score 3) of the anterior corners of the lumbar and cervical vertebrae are considered. Nonetheless, the reliability of mSASSS may be limited especially for those vertebrae corners scored as 1. In a recent report, a kappa analysis showed worse agreement on grade 1 lesions, which was relatively greater for syndesmophytes (0.163–0.559) and for ankylosis (0.48–0.95) (39). In the 1st prospective trial (7, 8), mSASSS was scored by a single observer (AW), suggesting the reliability of the scores might not be very high.

Data from the OASIS cohort also showed that the consistency of scores for individual vertebrae corners among different assessors was worse (21). In an earlier report, the mean progression rate per 2 years in the OASIS study was 1 mSASSS unit, but in the latest analysis a mean progression rate per year of 1 mSASSS unit was found (21, 40). These data imply that the reliability of changes in mSASSS over 2 years is not so good, especially for scoring by a sole assessor or for unknown time points.

Furthermore, observational studies and re-evaluations of a number of clinical trials had cast doubt on the scoring reliability of the radiographic sacroiliitis by individual investigators (41, 42).

### Radiographic Progression in AS I*s* Very Slow and Non-linear

In a retrospective hospitalized cohort of 146 TNFi naïve patients with AS, radiographic progression was not linear over 3.8 ± 1.7 years, and the mean rate of radiographic progression was 1.3 ± 2.5 mSASSS units each year (20). The OASIS study, with up to a 12-year prospective follow-up, revealed that mSASSS progressed very slowly from 11.6 ± 16.1 units at baseline to 24.5 ± 21.7 units after 12 years. The mean rate of radiographic progression was 0.98 mSASSS units every year (21). However, independent of symptom durations and follow-up times, variable rates of radiographic progression were seen within and across patients. Among the patients, individual mSASSS progression curves often alternate with periods of steep progression and relative quiescence (21).

Earlier trials with TNFi in AS patients have shown ineffective inhibition of radiographic progression compared to historical TNFi naïve patients (3), but recent observational studies with long-term follow-up have shown that TNFi can slow the rate of radiographic progression (23, 27, 43). The reasons for the different conclusions might be a more prolonged treatment and periods of observation, which may have detected slow changes. These data imply that 2-year treatment with NSAIDs is not sufficient to conclude reliably inhibitory effect of NSAIDs on spinal radiographic progression.

### Great Individual Variation of Radiographic Progression Exists in AS Patients

Long-term radiographic progression exhibits marked variability in AS patients. In a hospitalized cohort over 3.8 ± 1.7 years, a 4-fold greater rate of progression than the mean was found in 43% of patients while no progression was found in 23% of them (20). Over the entire follow-up period of 12 years in the OASIS cohort, nearly a quarter of patients showed no progression, a quarter showed rapid progression and half of the patients showed rates of progression of about 2 units per 2 years. In the first 2 years, approximately half of the patients did not exhibit significant mSASSS progression, whereas a quarter showed an mSASSS increase ≥ 5 units (21). These data imply that the small sample size of the studies of Haroon et al. (12) and Poddubnyy et al. (10) would weaken the validity of their conclusions.

## Conclusion

There is a paucity of studies that have focused on the therapeutic efficacy of NSAIDs to modify spinal radiographic progression in AS patients. Most studies have shown that continuous NSAIDs or high intake of NSAIDs does not delay radiographic progression, even in AS patients with elevated CRP levels. Several major drawbacks have been identified in the methodology of the studies performed to date, such as only 2 prospective randomized control trials, small numbers of patients in each cohort, and relatively brief periods of follow-up. So in both the ACR/SAA/SPARTAN 2015 recommendations for the treatment of AS (5) and the ASAS-EULAR 2016 management recommendations for AS (4), NSAIDs should only be prescribed to relieve the symptoms of AS patients rather than to delay structural damage.

## Author Contributions

J-RZ and S-MD conceived and conducted the study, analyzed and interpreted the data, and participated in drafting and revising the manuscript. D-DP made substantial contributions to the data analysis, data interpretation, and drafting of the manuscript. All the authors read and approved the final manuscript.

### Conflict of Interest

The authors declare that the research was conducted in the absence of any commercial or financial relationships that could be construed as a potential conflict of interest.
