# Supplementary material for: Non-steroidal Anti-inflammatory Drugs Are Unlikely to Inhibit Radiographic Progression of Ankylosing Spondylitis: A Systematic Review
Source: Front Med (Lausanne). 2019 Oct 4;6:214. doi: 10.3389/fmed.2019.00214 (PMC6788556; doi:10.3389/fmed.2019.00214)
Supplement: Supplementary file 1 [file Data_Sheet_1.PDF]

Search Strategy

The MEDLINE (via Pubmed), EMBASE (via Ovid) and Cochrane databases were searched using the following terms:

MEDLINE via Pubmed

1. Spondylitis, Ankylosing” [Mesh]
2. “Anti-Inflammatory drugs, Non-Steroidal” [Mesh]
3. “Disease Progression” [Mesh]
4. #1 and #2 and #3

EMBASE via Ovid

1. “ankylosing spondylitis”/exp OR ankylosing spondylitis
2. “non-steroidal anti-inflammatory agents”/exp “non-steroidal anti-inflammatory agents”
3. “Disease Progression”/exp “Disease Progression”
4. #3 AND #6 AND #9

The Cochrane Library

1. MeSH descriptor: [Spondylitis, Ankylosing] explode all trees
2. MeSH descriptor: [Anti Inflammatory Agents, Nonsteroidal] explode all trees
3. MeSH descriptor: [Disease progression] explode all trees
4. 1 AND #2 AND #3

Risk of Bias Assessment

Supplementary Table 1. The consensus risk of bias (RoB) from the two assessors are shown below. Domains with high, some concerns and low risk of bias are shown. Where the risk of bias is high or some concerns, the superscript letters refer to the specific reasons for the bias allocation, as detailed after the tables.

Domain / Study	randomization process	intended interventions	Missing outcome data	measurement of the outcome	selection of the reported result	Total RoB
Wanders 2005[7]	Low	Low	Some concerns[c]	Low	Low	Low
Kroon 2012[8]	Low	Low	Some concerns[c]	Low	Low	Low
Sieper 2016[9]	Low	Low	Some concerns[c]	Low	Low	Low
Poddubnyy 2012[10]	High [a]	Some concerns [b]	Low	Low	Low	Some concerns
Park 2019 [11]	High [a]	Some concerns [b]	Low	Low	Low	Some concerns

[a] Cohort study design lacking randomization

[b] Patients, physicians and people delivering the interventions were aware of the assigned intervention during the trial.

[c] More than 5% of the outcome data were unavailable.
